# Socioeconomic inequalities in hypertension in Kenya: a decomposition analysis of 2015 Kenya STEPwise survey on non-communicable diseases risk factors

**DOI:** 10.1186/s12939-020-01321-1

**Published:** 2020-12-02

**Authors:** Samwel Maina Gatimu, Thomas Wiswa John

**Affiliations:** 1 School of Economics, University of Nairobi, Nairobi, Kenya; 2 Medical Department, Mkinga District Council, Tanga, Tanzania

**Keywords:** Socioeconomic inequality, Hypertension, Kenya, NCDs, Inequalities, STEPwise, Risk factors

## Abstract

**Background:**

One in four Kenyans aged 18–69 years have raised blood pressure. Despite this high prevalence of hypertension and known association between socioeconomic status and hypertension, there is limited understanding of factors explaining inequalities in raised blood pressure in Kenya. Hence, we quantified the socioeconomic inequality in hypertension in Kenya and decomposed the determinants contributing to such inequality.

**Methods:**

We used data from the 2015 Kenya STEPwise survey for non-communicable diseases risk factors. We included 4422 respondents aged 18–69 years. We estimated the socioeconomic inequality using the concentration index (C) and decomposed the C using Wagstaff decomposition analysis.

**Results:**

The overall concentration index of hypertension in Kenya was − 0.08 (95% CI: − 0.14, − 0.02; *p* = 0.005), showing socioeconomic inequalities in hypertension disfavouring the poor population. About half (47.1%) of the pro-rich inequalities in hypertension was explained by body mass index while 26.7% by socioeconomic factors (wealth index (10.4%), education (9.3%) and paid employment (7.0%)) and 17.6% by sociodemographic factors (female gender (10.5%), age (4.3%) and marital status (0.6%)). Regional differences explained 7.1% of the estimated inequality with the Central region alone explaining 6.0% of the observed inequality. Our model explained 99.7% of the estimated socioeconomic inequality in hypertension in Kenya with a small non-explained part of the inequality (− 0.0002).

**Conclusion:**

The present study shows substantial socioeconomic inequalities in hypertension in Kenya, mainly explained by metabolic risk factors (body mass index), individual health behaviours, and socioeconomic factors. Kenya needs gender- and equity-focused interventions to curb the rising burden of hypertension and inequalities in hypertension.

**Supplementary Information:**

The online version contains supplementary material available at 10.1186/s12939-020-01321-1.

## Background

Low- and middle-income countries (LMICs) including Kenya bear the largest burden of non-communicable diseases (NCDs) [1]. High blood pressure is one of the risk factors of NCDs [2]. It affects about a third of the global population and causes an estimated 7.6 million premature deaths [1]. In Kenya, a quarter of the population aged 18–69 years is estimated to be hypertensive [3] and a half pre-hypertensive [4]. The prevalence of hypertension is higher among women and urban residents than among men and rural residents [5]. Hypertensive disease is associated with a high burden of out of pocket expenditure to patients and family with an annual cost of US$477 and contributing to 59% of the catastrophic healthcare costs [6].

Hypertension is associated with socioeconomic status [7–11]. Low socioeconomic status is associated with a high prevalence of hypertension, and untreated and uncontrolled hypertension in LMICs [7–11]. However, this is not a consistent finding. For example, in Kenya, a study among urban adults found a high prevalence of hypertension among the richest individuals [12] while others studies have shown a high prevalence among the poor individuals [10, 11]. A different study explaining the association between socioeconomic position and hypertension in Kenya found that education is not associated with hypertension but material resources were positively associated but not mediated by smoking and alcohol [13].

Furthermore, studies on socioeconomic inequalities in hypertension have also shown that both pro-poor and pro-rich inequalities exist in hypertension [9, 14, 15]. In Kenya, however, despite a high prevalence of hypertension, studies have shown the existence of social gradient in hypertension among different populations [5, 12, 16] but the extent and pattern of inequalities and potential factors explaining the socioeconomic inequalities in hypertension across wealth distribution are not well understood. Understanding inequalities in hypertension is key to inform interventions to prevent hypertension and reduce premature mortality from hypertension by 25% by 2025 in line with the global [2] and national targets [17]. Therefore, this study quantified the socioeconomic inequality in hypertension in Kenya and decomposed the determinants contributing to such socioeconomic inequality.

## Methods

### Data source and study population

The study used data from the nationwide 2015 Kenya STEPwise survey for non-communicable diseases and injury risk factors among adults aged 18–69 years in Kenya [3]. The survey used a three-stage cluster sample design: selection of 200 equally distributed urban and rural areas clusters, 30 sampled households from the selected clusters and random sampling of one member from all the listed households’ members in each household. The STEPwise survey targeted population based on age-sex group, and involved individuals aged 18–69 years [3]. Out of the 4654 households that gave consent, 4500 individuals consented to participate in the survey yielding a 95% response rate. Structured questionnaires based on WHO STEPwise approach to chronic disease risk factors—demographic and behavioural information (Step 1), anthropometric (Step 2) and biochemical measurements (Step 3)—was used [3]. Personal digital assistant loaded with the eSTEPS questionnaire were used by the trained data collectors to record data. Various quality assurance measures were put in place including regular checks of the collected data for consistency by a quality assurance team. The Kenya Medical Research Institute Ethics Review Committee approved the survey and all eligible participants gave written informed consent [3]. The deidentified data used in this study were accessed from the Kenya National Bureau of Statistics based on a data access agreement [18]. More details of the survey are published elsewhere [3].

### Measures

#### Outcome variable

Hypertension, our main outcome variable was defined as “a systolic blood pressure of ≥140 mmHg and/or diastolic blood pressure of ≥90 mmHg on two separate occasions or self-reported use of blood pressure medication and/or previous diagnosis by a healthcare provider” [19, 20]. A binary variable was generated where 0 was normal while 1 was hypertension.

#### Socioeconomic measure

Wealth index was used as the measure of socioeconomic position and living standards of the respondents. The wealth index was computed based on the data on household assets using principal component analysis (PCA) [21]. PCA is the standard method to calculate wealth index used in most demographic and health surveys [22, 23]. Key household assets variables assessing ownership of infrastructures and amenities such as source of drinking water, type of toilet, type of house’s floor, walls and roof, cooking fuels and availability of electricity were included in the analysis (Supplementary Table 1) [3, 22, 23]. An index was created based on the sum of all weights of included variables, which were generated by PCA after testing and fulfilling all the three assumption (Bartlett’s test, Kaiser-Meyer-Olkin measure of sampling adequacy and determinants of matrix correlation) [21, 24]. The resulting continuous-scale wealth index was later categorised into five quintiles (1st quantile – poorest, 2nd quantile – poorer, 3rd quantile – middle, 4th quantile – richer and 5th quantile – richest) [24].

#### Social determinants of hypertension

The social determinants of hypertension included demographic (age, sex, marital status), socioeconomic (education, occupation, wealth index), anthropometric (body mass index), health behaviours (physical activity, current smoking use, current alcohol use, and fruits and vegetable intake) and community (region and residence) variables (Table 1).

**Table 1:** Social determinants of hypertension

Variable	Operational definition	Source
Age in years	Age of the respondents at the time of the interview was categorised into 18–29; 30–39; 40–49; 50+ years	[25–28]
Sex	Male or female	[25–28]
Marital status	In-a-union (married or cohabiting); Not-in-a-union (Never married, divorced, separated, or widowed)	[5, 12, 25]
Education levels	No formal; primary incomplete; primary complete; secondary+	[12, 25, 29]
Residence	Urban or rural areas	[5, 25]
Region	Nairobi, Central, Eastern, Western, Nyanza, Coast, Rift Valley, North Eastern	[25]
Occupation	Unemployed, self-employed, paid employment	[12, 25, 29]
Physical activity levels	Data collected based on the WHO Global Physical Activity questionnaire (GPAQ) was analysed according to the GPAQ analysis guide [30] and classified into: low (< 600 metabolic equivalent of task, METS, minutes per week), moderate (600–1500), and high (> 1500 METS minutes per week)	[5, 12, 25]
Current smoker	Current use of tobacco, tobacco products and smokeless cigarette was classified as Yes or No	[14, 25, 31, 32]
Current alcohol use	Current use of any type of alcohol was classified as Yes or No	[14, 25, 33]
Fruits & vegetable intake	Sufficient (≥5 servings); insufficient (< 5 servings)	[25, 27]
Body mass index	Undernutrition (≤18.5 kg/m^2^), Normal (18.5–24.9 kg/m^2^), Overweight (≥25–29.9 kg/m^2^), Obese (≥30 kg/m^2^)	[25, 28, 34–36]

### Statistical analysis

Sample characteristics and prevalence of hypertension were described using frequencies and percentages. Stata 13.1 was used to perform all statistical analyses that were adjusted for the stratified sampling design of the survey.

Socioeconomic inequalities in hypertension in Kenya was quantified using concentration index (C) and depicted using concentration curves. C is a ‘measure of inequality in the distribution of health outcome across the wealth distribution’, reflects the experience of the population as the whole and is sensitive to the change in the distribution of the population across social-economic groups and can be decomposed [37]*.* C ranges from − 1 and + 1 and C = 0 shows perfect equality while C < 0 shows hypertension is disproportionately concentrated among the poor (pro-rich inequality). It was computed based on the proportion of hypertension by the ranked wealth distribution as follows [37]:

1 $$ \mathrm{C}=\frac{2}{n\mu}\ \sum \limits_{i=1}^n{h}_i{R}_i-1 $$

where *n* is the number of people, *μ* is the overall mean/proportion of *h*; *h*_*i*_ is hypertension in *i*^*th*^ person; *R*_*i*_ is the *i*^*th*^ person ranked by wealth index from the poorest to richest [37]. Hypertension is a binary variable, and the C was normalised by dividing the C by 1–***μ*** [38, 39]. We used the Wagstaff-type decomposition analysis of the concentration index [40] to examine the contribution of social determinants of inequality in hypertension. The method has been internationally accepted and has widely been used [41–45] to decompose inequalities across the full distribution of wealth index rather than between the richest and the poorest [37]. It allows for the overall concentration index to be decomposed into contributions of social determinants of health, in which each social determinant’s contribution is obtained by multiplying the sensitivity of the outcome variable with respect to that determinant and the degree of wealth-related inequality in that factor [37]. It also allows decomposition of the changes in the elasticities of health outcome with respect to these social determinants [37].

To decompose, we first considered the linear additive regression model for the outcome variable (y) of an individual *i* as follow:

2 $$ {\mathrm{y}}_i\kern0.5em =\kern0.5em a\kern0.5em +\Sigma {\mathrm{k}\upbeta}_{\mathrm{k}}{\mathrm{x}}_{\mathrm{k}\mathrm{i}}\kern0.5em +\kern0.5em {\varepsilon}_i $$

where the concentration index of health outcome (y) can be written as:

3 $$ {\mathrm{C}}_{\mathrm{Normalized}}=\frac{\mathrm{C}}{1-\mu }=\frac{\sum \mathrm{k}\left(\frac{\upbeta_{\mathrm{k}}{\overline{\mathrm{x}}}_{\mathrm{k}}}{\mu}\right){\mathrm{C}}_{\mathrm{k}}}{1-\mu }+\frac{\mathrm{GC}\upvarepsilon /\mu }{1-\mu } $$

where *μ* is the mean of hypertension, ${\overline{x}}_k$ is the mean of *xk*, *C*_*k*_ is the concentration index of *xk*, and *GCε* (residual) is the generalized concentration index for the error term (*ε*). C, therefore, is the sum of the two components – the concentration index of the explanatory variables weighted by the elasticity of *y* $\left({\beta}_k{\overline{x}}_k/\upmu \right)$ which is the explained part and residual part (GCε/ μ) which is the unexplained part [37]. Hypertension, the outcome variable, is non-linear hence a non-linear Probit model was used to estimate the marginal effects (*β*_*k*_) of each determinant [37]. The marginal effects were used to calculate the contribution of *k* determinants. A negative absolute contribution was interpreted as supporting effect of inequality that favours the rich, to the disadvantage of poor and vice versa. The absolute contributions were adjusted by dividing the absolute contribution of each determinant by the total explained part that make contributions to the same direction of the concentration index.

## Results

### Sample characteristics

Out of the 4500 eligible participants, 4422 respondents were included in the analysis after excluding 15 respondents aged below 18 years or above 69 years and 63 who had missing or incorrect data on blood pressure. A majority of study participants were female (51.4%), aged 18–29 years (46.3%), lived in rural areas (61.4%), in-a-union (65.5%) and unemployed (40.2%). A quarter of the respondents (25.3%) currently used alcohol, 12.5% were current smokers, 10.8% had low physical activity and 11.2% were obese. Table 2 outlines the respondents’ characteristics and the prevalence of hypertension according to the sociodemographic, socioeconomic, behavioural, and anthropometric characteristics.

**Table 2:** Respondents’ characteristics and prevalence of hypertension by respondents’ characteristics

Categories	Characteristics	Sample (***N*** = 4422)		Prevalence of hypertension	
		n	%	n	% [95% CI]
**Sociodemographic variables**	**Sex**			1280	25.8 [23.4, 28.3]
	Male	1761	48.6	533	28.1 [24.7, 31.8]
	Female	2661	51.4	747	23.6 [21.2, 26.2]
	**Age, years**				
	18–29	1472	46.3	228	14.7 [12.0, 17.9]
	30–39	1230	23.2	275	23.1 [19.0, 27.7]
	40–49	780	15.5	275	36.9 [31.4, 42.8]
	50+	940	15.0	502	52.8 [48.4, 57.2]
	**Marital status**				
	Not-in-a-union	1415	34.5	420	22.1 [18.7, 25.9]
	In-a-union	3006	65.5	860	27.7 [24.9, 30.8]
**Socioeconomic variables**	**Education**				
	No formal	745	12.6	215	24.5 [19.3, 30.7]
	Primary incomplete	1080	23.3	289	23.3 [20.2, 26.8]
	Primary complete	1398	32.7	419	27.5 [23.7, 31.7]
	Secondary+	1199	31.4	357	26.3 [21.7, 31.5]
	**Occupation**				
	Unemployed/Unpaid	1849	40.2	497	22.2 [19.3, 25.5]
	Self-employment	1757	40.0	525	26.2 [22.9, 29.9]
	Paid employment	816	20.8	258	31.8 [26.1, 38.0]
	**Wealth index (quintiles)**				
	1 – Poorest	885	23.6	297	28.2 [22.7, 34.4]
	2	884	20.3	272	29.1 [24.8, 33.8]
	3	885	19.6	263	24.1 [20.0, 28.7]
	4	884	18.7	237	26.4 [22.1, 31.3]
	5 – Richest	884	17.8	211	20.1 [16.6, 24.1]
**Health behaviours**	**Current smoking**				
	No	3916	87.5	1128	25.6 [23.4, 28.0]
	Yes	506	12.5	152	26.9 [20.9, 33.8]
	**Current alcohol use**				
	No	3504	74.7	971	24.0 [21.8, 26.5]
	Yes	911	25.3	308	31.1 [27.9, 35.8]
	**Fruits & vegetable intake**				
	Enough	576	11.2	166	25.0 [20.3, 30.4]
	Not enough	3822	88.8	1114	25.9 [23.3, 28.6]
	**Physical activity**				
	High	3236	75.1	927	25.6 [23.9, 28.6]
	Moderate	662	14.1	188	25.1 [20.2, 30.7]
	Low	524	10.8	165	27.7 [21.2, 35.3]
**Metabolic risk factor**	**Body mass index**				
	Normal	2250	56.3	568	23.2 [20.7, 25.8]
	Undernutrition	683	15.8	136	16.7 [13.2, 20.8]
	Overweight	775	16.7	286	33.4 [28.3, 38.9]
	Obese	550	11.2	266	45.8 [40.6, 51.1]
**Community variables**	**Residence**				
	Rural	2268	61.4	647	26.5 [23.8, 29.4]
	Urban	2154	38.6	633	24.7 [20.5, 29.4]
	**Regions**				
	Rift Valley	1325	24.6	355	24.9 [21.1, 29.1]
	Eastern	767	14.0	258	30.3 [25.7, 35.3]
	Nyanza	569	12.5	149	23.9 [19.0, 29.7]
	Coast	533	9.3	140	20.7 [14.8, 28.2]
	Central	507	12.4	208	38.5 [30.4, 47.3]
	Western	408	10.3	112	26.7 [23.9, 29.7]
	North Eastern	250	4.9	43	16.0 [9.8, 25.0]
	Nairobi	63	12.1	15	18.5 [11.2, 29.1]

Note: All the percentages are weighted

### Prevalence of hypertension

The overall weighted prevalence of hypertension was 25.8% (95% CI: 23.4–28.3%) with a higher prevalence among men than women (28.1% vs 23.6%). The prevalence of hypertension was higher among those who were 50+ years (52.8%), paid employment (31.8%), obese (45.8%) and overweight (33.4%) and from the central region (38.5%). The prevalence of hypertension was also higher among the poorest (28.2%) and poorer (29.1%) compared to the richest (20.1%) but did not show a clear wealth-related gradient (Table 2).

### Socioeconomic inequality in hypertension

**Fig. 1:**
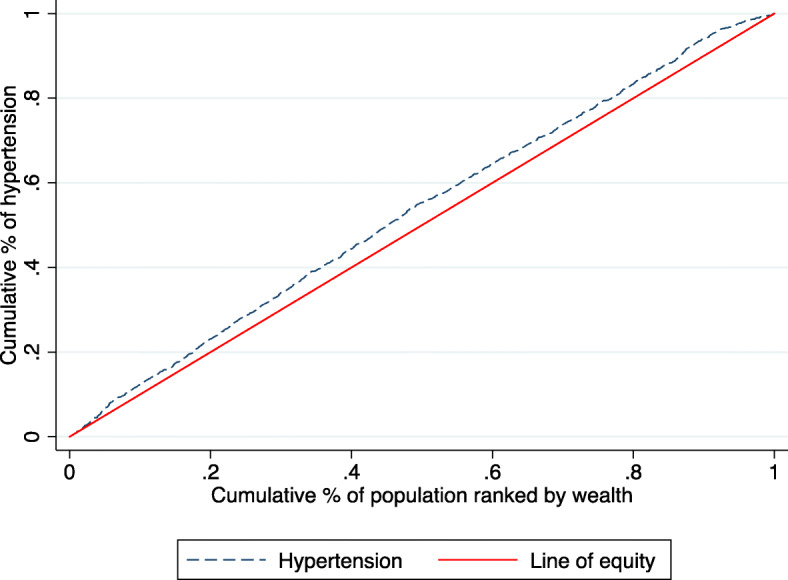
Concentration curve (C = − 0.081, 95% CI: − 0.138, − 0.024; *p* = 0.005)

**Fig. 2:**
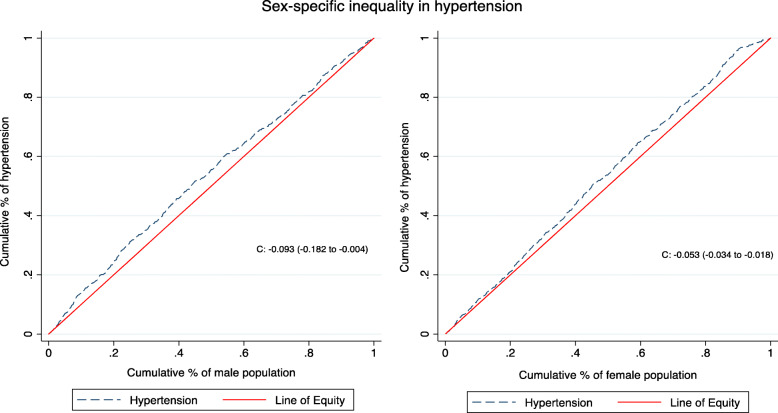
Concentration curve for male (C = − 0.094, 95% CI: − 0.182, − 0.006; *p* = 0.036) and female (C = − 0.053, 95% CI: − 0.125, − 0.018; *p* = 0.142) population

Figures 1 and 2 shows that the concentration curve lay above the line of equality indicating a pro-rich inequality in hypertension disfavouring the poor, with an overall concentration index of hypertension of − 0.08 (95% CI: − 0.14, − 0.02; *p* = 0.005). The social determinants included in our model explained 99.7% of the estimated socioeconomic inequality in hypertension in Kenya with a small non-explained part of the inequality (− 0.0002).

Table 3 presents results of the decomposition analysis that revealed that about half (47.1%) of the pro-rich inequalities in hypertension is explained by the body mass index. Slightly more than a quarter (26.7%) of the estimated inequalities in hypertension is explained by socioeconomic factors (paid employment (7.0%), education (9.3%) and wealth index (10.4%)) while sociodemographic factors (female gender (10.5%), age (4.3%) and marital status (0.6%)) explained 15.4% of the observed inequality. Lastly, the regions explained 7.1% of the estimated inequality with the Central region alone explaining 6.0% of the observed inequality while individual health behaviour explained only 3.7% (smoking (2.3%) and alcohol use (1.4%)).

**Table 3:** Summary of decomposition analysis

Category	Characteristics	Coeff.	Elasticity	C	Cont. to C	%	Adjusted %^**a**^
**Sociodemographic variables**	**Female gender** (Ref: male)	− ***0.057***	−0.132	0.122	−0.016	**20.1**	**10.5**
	**Married** (Ref: unmarried)	0.009	0.024	−0.040	−0.001	**1.2**	**0.6**
	**Age, years (Ref:** 18−29 years)						**4.3**
	30–39	**0.071**	0.077	−0.004	−0.0003	0.4	**0.2**
	40–49	**0.191**	0.131	−0.049	− 0.006	7.9	**4.1**
	50+	**0.386**	0.318	0.123	0.039	−48.8	
**Socioeconomic variables**	**Education** (Ref: No formal)						**9.4**
	Primary incomplete	−0.015	−0.014	0.255	−0.004	4.7	**2.5**
	Primary complete	0.045	0.055	−0.117	−0.006	8.0	**4.2**
	Secondary+	0.007	0.007	−0.594	−0.004	5.1	**2.7**
	**Occupation** (Ref: Unemployed)						
	Self-employment	−0.001	−0.002	−0.033	0.0001	−0.1	
	Paid employment	0.031	0.022	−0.475	− 0.011	13.3	**7.0**
	**Wealth Index** (Ref: Poorest)					−**0.8**	**11.7**
	Poorer	0.023	0.018	0.499	0.009	11.2	**5.9**
	Middle	−0.018	− 0.014	0.0002	0.000	0.0	
	Richer	0.008	0.023	−0.500	0.003	−4.0	
	Richest	−0.009	0.007	−1.000	− 0.007	8.5	**4.5**
**Health behaviours**	**Current smokers** (Ref: No)	−0.028	−0.013	0.281	− 0.004	**4.4**	**2.3**
	**Current alcohol use** (Ref: No)	**0.068**	0.054	−0.038	−0.002	**2.6**	**1.4**
	**Insufficient fruits/vegetable intake**	0.024	0.080	0.023	0.002	−**2.3**	
	**Physical activity** (Ref: High)						
	Moderate	−0.009	−0.005	−0.156	0.001	−1.0	
	Low	0.032	0.015	0.084	0.001	−1.5	
**Metabolic risk factor**	**Body mass index** (Ref: Normal)						**47.1**
	Undernutrition	−**0.089**	−0.055	0.386	−0.021	26.8	**14.1**
	Overweight	**0.090**	0.064	−0.227	− 0.015	18.1	**9.5**
	Obese	**0.200**	0.100	−0.359	− 0.036	44.9	**23.5**
**Geographical factors**	**Urban residence** (Ref: rural)	−0.013	−0.024	−0.388	0.009	−**11.6**	
	**Regions** (Ref: Rift Valley)					**11.6**	**7.1**
	Eastern	0.021	0.014	− 0.011	−0.0002	0.2	**0.1**
	Nyanza	−0.016	−0.008	0.076	− 0.006	0.7	**0.4**
	Coast	−0.050	− 0.023	0.013	−0.0003	0.4	**0.2**
	Central	***0.063***	0.028	−0.325	−0.009	11.4	**6.0**
	Western	−0.008	−0.003	−0.043	0.0001	−0.2	
	North Eastern	−0.005	−0.001	0.644	−0.007	0.8	**0.4**
	Nairobi	−0.067	−0.004	−0.541	0.002	−2.5	
	Concentration index			−0.081			
	Standard error			0.029			
	Residuals			−0.0002			
	95% CI		−0.138 to − 0.024				

Bold: *p* < 0.05. *Coeff*. coefficient (marginal effects); *C* concentration index; *Cont* to C: contribution to concentration index; *%* percentage contribution; *Ref* reference category; *SE* standard error; ^**a**^ The absolute contribution of each determinant was divided by the total explained portion that make contributions to the same direction of the concentration index.

Respondents who were middle-aged, unmarried, urban residents, with primary school and higher education, moderately physically active, current alcohol users, overweight and obese and from Nairobi, Western, Central and Eastern regions were mainly concentrated among the poor populations (indicated by the negative concentration indices) and were likely to be hypertensive (indicated by the coefficients) (Table 3).

## Discussion

Our study revealed a pro-rich inequality in hypertension in Kenya, disfavouring poor individuals. The inequality is explained by body mass index, socioeconomic (wealth index, occupation, and education), sociodemographic (gender, age, and marital status) factors, regions and individual health behaviours (current history of alcohol use and smoking). The prevalence of hypertension reported in the current study is similar to previous studies conducted in Kenya [12, 16, 46–48]. Similarly, the high prevalence of hypertension among men and older adults compared to women and younger adults have also been established [12, 16, 46–48].

Similar to other studies in LMICs [8, 29, 49, 50], our findings indicate the presence of inequalities in hypertension disfavouring the poor population. The magnitude of the inequalities in our study (C: − 0.08) is lower compared to that of Iran (C: − 0.15) [29, 50] and among rural residents in Bangladesh (C: − 0.20) [29, 50] despite an almost similar hypertension prevalence reflecting the varying levels of inequalities. However, our findings differ from the pro-poor inequality in high blood pressure reported in a study among women of reproductive age in sub-Saharan Africa [14]. The study reported cumulative inequality for sub-Saharan Africa and did not compute country-specific inequalities which could explain the difference. Nevertheless, our study shows the size of inequality to be lower among women than men (C: − 0.05 vs − 0.09) and the hazardous effect of hypertension concentrated among the underprivileged populations who are poor.

Body mass index was the largest independent contributor to the inequality in hypertension explaining about half of the inequality. Our study shows that almost one in three individuals were overweight or obese and had some of the highest prevalence of hypertension. Obesity and overweight are known risk factors for hypertension [28, 34–36] and independently contributed to 23.5 and 9.5% of the observed inequality. About 40% of the obese participants in our study belonged to the poorest wealth quintile. Obesity/overweight increases an individual risk of hypertension especially among individual belonging to the poorest group [12, 16, 35]. The high burden of overweight and obesity in Kenya could be attributed to the rapid urbanisation, economic development, and the related unhealthy behaviours such as consumption of energy dense processed food and sedentary lifestyles [51]. As an ecological study in Kenya has showed, there is a positive association between the rise of NCDs including hypertension in Kenya and the increase in per capita gross domestic product, urbanisation, physical inactivity and consumption of high dense processed foods such as cooking oil and wheat [52].

Surprisingly, undernutrition independently contributed 14.1% of the observed inequality, which could reflect the high prevalence of hypertension (16.7%) among the 16% of our study population who were undernourished. High prevalence of hypertension has previously been reported among undernourished individuals [53]. We hypothesise that the life course approach showing the relationship between childhood/adulthood malnutrition and hypertension could help explain this finding. Malnutrition limits renal development resulting in kidney malfunction in adulthood and eventually hypertension [54, 55]. However, we studied adult individuals some of whom were already malnourished and could not ascertain the timing of the occurrence of malnutrition and any causal linkage to their hypertension. This calls for further evaluation to establish the causal relationship between adult undernutrition and hypertension with a focus on the poor populations.

Occupation, education, and wealth index are the socioeconomic factors contributing to slightly more than a quarter of the observed inequalities in hypertension. Similarly, these factors also explained inequality in hypertension in Iran [29]. In this study, individuals in formal paid employment were more concentrated among the poor and had a relatively high prevalence of hypertension compared to the unemployed and self-employed individuals. In Kenya, studies have shown that casual workers and individuals on formal employment have increased odds of hypertension [12] which could be attributed to sedentary lifestyle. Our findings also revealed that individuals with low education level (incomplete or complete primary educations) were more likely to be hypertensive and poor. Low education level is associated with the risk of developing hypertension [16, 27, 28]. However, among older adults in Kenya, education was found not be associated with hypertension [13]. Despite this, educated individuals have a better awareness of hypertension and its preventive strategies compared to the uneducated [10, 11]. Wealth index explained one-tenth of the observed inequality in hypertension with almost equal individual contribution from both the richest and poorer wealth quintiles. Our findings show a high prevalence of hypertension among individuals in the poorest wealth quintile, which is inconsistent with previous studies [12, 14]. For example, one of the studies in the urban slums in Nairobi Kenya found that individuals in the richest wealth quintile had the highest prevalence of hypertension and were at an increased odd of being hypertensive [12]. This could be due to the differences in study population. However, we hypothesize the poorest individuals faces several financial barriers, which hinder access to health services for control and treatment of hypertension [56] due to huge out of pocket expenditure [6, 57]. In addition, despite the hypertension screening and early diagnosis being key in averting hypertension, it is likely that individuals in the richest wealth quintile are screened more for high blood pressure than the poorest individuals. Among urban poor populations in Kenya, increased wealth may contribute to unhealthy behaviours such as consumption of energy dense processed food and sedentary lifestyles [12].

Socio-demographic factors explained about 15% of the observed inequality. Specifically, gender had a substantial contribution to the inequality with men having a significantly higher prevalence of inequalities in hypertension than women. Previous studies have observed gender disparities in hypertension [26–28], which have been attributed to biological [58] and health behavioural factors [59]. In this study, a further gender-specific decomposition shows that the differences in body mass index, education, employment, and the region could explain the gender disparities in hypertension (Supplementary Table 2). Age, especially 40–49 years also contributed to the observed inequality in hypertension. Adults aged 35 years and above have increased the risk of hypertension in Kenya [12], which is supported by our finding that a third of these adults are hypertensive. Overall, older adults in Kenya have increased odds of multiple NCD risk factors [60] hence increased risk of hypertension. Moreover, the current study shows substantial inequality in hypertension for the older and poor population. These findings call for gender-focused approaches in prevention, treatment, and control of hypertension in Kenya.

Regional differences mainly attributed to Central region contributed to 7.1% of the observed inequalities in hypertension. The central region had the highest prevalence of hypertension. Its main inhabitants, the Kikuyu ethnic groups, has high prevalence hypertension [61] and cardiometabolic markers [60]. It is also one of the most unequal regions in Kenya [62] with a large population of older adults (7%) and high prevalence of alcohol use and smoking. At local level, the high prevalence of hypertension has been speculated to results from poor dietary practices such as high consumption of carbohydrates and sedentary lifestyles [63] and poor awareness on hypertension [64].

Behavioural risk factors contributing to the inequalities in hypertension were current history of smoking and alcohol use. Alcohol use and smoking are associated with increased risk of hypertension [27, 34, 65, 66]. The prevalence of smoking [14, 31, 32] and alcohol use [14, 33] was high among individuals in the poorest wealth quintile. These poor individuals are also likely to be hypertensive hence contribute to hypertension inequality in Kenya. To address the challenges of smoking and alcohol use, Kenya has through legislation banned advertisement of tobacco and its products, limited the number of cigarettes sold, criminalised the sale of illegal alcohol and increased taxes on alcohol and tobacco and its products.

### Strengths and limitations

To our knowledge, this is the first study in Kenya quantifying and explaining the inequalities in hypertension. One of the strengths of the study is the use of data collected based on the standardised WHO STEPwise approach from a large nationally representative sample making our finding generalisable to Kenya and comparable to studies using similar approach. Also, the study variables included explained the observed inequalities with minimal residual. However, the study had some limitations. First, the data used was cross-sectional and hence causal inference could not be made. Second, the blood pressure measurements were taken in one visit which could have resulted in an overestimation of the prevalence. Third, there was a potential for recall bias in self-reported variables such as physical activity, tobacco and alcohol use and fruits and vegetable consumption, which may have over- or underestimated their prevalence. Fourth, the study included some explanatory variables that are closely related such as wealth, education, and occupation in the decomposition analysis. Inclusion of all these variables may have resulted in the underestimation of the full effect of either of the variables and may have ignored the potential causal hierarchy among the variables, for example, education being a driver for wealth and occupation.

## Conclusion

The present study shows pro-rich inequality in hypertension, which is mainly explained by individual health behaviour, socioeconomic and sociodemographic factors. These findings are particularly important considering that more than half of the Kenyan population, aged 18–69 years are pre-hypertensive [48, 67]. We call for gender- and equity-focused interventions, as proposed in the national strategy for non-communicable diseases to curb the rising burden of hypertension and address the inequalities in hypertension. Importantly, the study findings highlight the significant contribution of obesity/overweight in hypertension inequalities, which calls for further research and investments in measures to curb obesity such as taxes on highly processed foods, sweetened beverages and promoting physical activity.

## Supplementary information

Additional file 1: **Table S1.** Variables included in the calculation of wealth index. Table S2. Summary of decomposition analysis for male and female.

## Data Availability

Data used in this study can be accessed based on the Kenya National Bureau of Statistics data access agreement on http://statistics.knbs.or.ke/nada/index.php/catalog/90

## Abbreviations

C: Concentration index
MET: Metabolic Equivalent of Task
NCDs: Non-communicable diseases
US: United States
WHO: World Health Organization
SE: Standard error
