# Supplementary material for: Socioeconomic inequalities in hypertension in Kenya: a decomposition analysis of 2015 Kenya STEPwise survey on non-communicable diseases risk factors
Source: Int J Equity Health. 2020 Dec 2;19:213. doi: 10.1186/s12939-020-01321-1 (PMC7709247; doi:10.1186/s12939-020-01321-1)
Supplement: Supplementary file 1 — Table S1. Variables included in the calculation of wealth index. Table S2. Summary of decomposition analysis for male and female. [file 12939_2020_1321_MOESM1_ESM.docx]

**Supplementary Tables**

**Supplementary Table 1. Variables included in the calculation of wealth index**

| **Variable** | **Response options** |
| --- | --- |
| Source of drinking water | Piped into dwelling, piped into compound, piped to neighbour, piped to kiosk, public tap, borehole, protected dug well, unprotected dug well, protected spring water, unprotected spring water, rainwater collection, tanker-truck, cart with small tank/drum, surface water, bottled water , other |
| Type of toilet | Flush to sewer, flush to septic tank, flush to pit latrine, flush to somewhere else, flush to unknown, ventilated improved pit latrine, pit latrine with slab, open pit latrine, composting toilet, bucket, hanging latrine, no facility |
| Type of floor | earth/sand, dung, wood planks, parquet or polished wood, vinyl or asphalt strips, ceramic tiles, cement, carpet |
| Type of roof | No roof, grass/thatch, dung/mud, iron sheet, tin cans, asbestos sheet, concrete, tiles |
| Type of wall | No walls, cane/palm/trunks, dirt, bamboo with mud, stone with mud, uncovered adobe, plywood, cardboard, reused wood, cement, stone with lime/cement, bricks, cement blocks, covered adobe, wood planks/shingles, others |
| Type of cooking fuel | Electricity, LPGAs, Natural gas, biogas, kerosene, coal, charcoal, wood, straw/shrubs/grass |
| Use of electricity | Yes, No |
| Possession of radio | Yes, No |
| Possession of tv | Yes, No |
| Possession of refrigerator | Yes, No |
| Possession of Washing machine | Yes, No |
| Availability of landline phone | Yes, No |
| Possession of mobile phone | Yes, No |
| Possession of bicycle | Yes, No |
| Possession of motorcycle/ scooter | Yes, No |
| Possession of watch | Yes, No |
| Possession of computer | Yes, No |
| Number of boats with motor | 1, 2, etc |
| Possession of car/ truck | Yes, No |
| Possession of animal drawn cart | Yes, No |
| Type household dwelling | Own, Rent, Squatter/stay for free/others |
| Help from employed person | Yes, No |
| Agricultural land ownership | Yes, No |
| Livestock ownership | Yes, No |

**Supplementary Table 2**. Summary of decomposition analysis for male and female

| **Characteristics** |  |  | **Male** |  |  |  |  |  |  | **Female** |  |  |  |
| --- | --- | --- | --- | --- | --- | --- | --- | --- | --- | --- | --- | --- | --- |
|  | **Coeff.** | **Elasticity** | **C** | **Cont. to C** | **%** | **Adjusted %** |  | **Coeff.** | **Elasticity** | **C** | **Cont. to C** | **%** | **Adjusted %** |
| **Age, years** (Ref: 18−29 years) |  |  |  |  |  | **1.5** |  |  |  |  |  |  | **5.6** |
| 30−39 | 0.0444 | 0.0457 | 0.0015 | 0.0001 | -0.1 |  |  | 0.0967 | 0.1111 | -0.0033 | -0.0004 | 0.7 | 0.2 |
| 40−49 | 0.1325 | 0.0900 | -0.0329 | -0.0030 | 3.2 | 1.5 |  | 0.2547 | 0.1799 | -0.0543 | -0.0098 | 18.4 | 5.4 |
| 50+ | 0.3091 | 0.2314 | 0.1102 | 0.0255 | -27.4 |  |  | 0.4646 | 0.4213 | 0.1284 | 0.0541 | -101.9 |  |
| **Married** (Ref: Unmarried) | 0.0335 | 0.0816 | -0.0648 | -0.0053 | **5.7** | 2.7 |  | 0.0288 | 0.0825 | -0.0238 | -0.0020 | **3.7** | 1.1 |
| **Education** (Ref: No formal) |  |  |  |  |  |  |  |  |  |  |  |  |  |
| Primary incomplete | 0.0809 | 0.0673 | 0.3045 | 0.0205 | -22.0 |  |  | -0.0399 | -0.0425 | 0.2186 | -0.0093 | 17.5 | 5.2 |
| Primary complete | 0.1442 | 0.1660 | 0.0672 | 0.0112 | -12.0 |  |  | 0.0225 | 0.0297 | -0.2367 | -0.0070 | 13.2 | 3.9 |
| Secondary+ | 0.0996 | 0.1251 | -0.5592 | -0.0700 | 75.2 | 36.1 |  | -0.0078 | -0.0072 | -0.6173 | 0.0045 | -8.4 |  |
| **Occupation** (Ref: Unemployed) |  |  |  |  |  |  |  |  |  |  |  |  |  |
| Self-employment | -0.0025 | -0.0042 | 0.1436 | -0.0006 | 0.7 | 0.3 |  | 0.0009 | 0.0014 | -0.1318 | -0.0002 | 0.3 | 0.1 |
| Paid employment | 0.0075 | 0.0076 | -0.4229 | -0.0032 | 3.5 | 1.7 |  | 0.0756 | 0.0376 | -0.5241 | -0.0197 | 37.2 | 11.0 |
| **Wealth** (Ref: Richest) |  |  |  |  |  | **23.6** |  |  |  |  |  |  |  |
| Poorest | 0.0220 | 0.0173 | -0.4001 | -0.0069 | 7.4 | 3.6 |  | 0.0138 | 0.0109 | -0.5633 | -0.0061 | 11.6 | 3.4 |
| Poorer | -0.0534 | -0.0369 | 0.1276 | -0.0047 | 5.1 | 2.4 |  | 0.0117 | 0.0101 | -0.0850 | -0.0009 | 1.6 | 0.5 |
| Middle | -0.0045 | -0.0031 | 0.5987 | -0.0018 | 2.0 | 0.9 |  | 0.0198 | 0.0173 | 0.4340 | 0.0075 | -14.2 |  |
| Richer | -0.0555 | -0.0322 | 1.0006 | -0.0322 | 34.6 | 16.6 |  | 0.0422 | 0.0401 | 1.0004 | 0.0401 | -75.5 |  |
| **Current smoking** (Ref: No) | -0.0242 | -0.0193 | 0.2668 | -0.0052 | **5.5** | 2.7 |  | -0.0123 | -0.0022 | 0.6532 | -0.0014 | **2.7** | 0.8 |
| **Current alcohol use** (Ref: No) | 0.0868 | 0.1223 | 0.0058 | 0.0007 | -0.8 |  |  | 0.0409 | 0.0141 | 0.0555 | 0.0008 | -1.5 |  |
| **Insufficient fruits/vegetable intake** | 0.0701 | 0.2158 | 0.0257 | 0.0056 | -6.0 |  |  | -0.0257 | -0.0954 | 0.0164 | -0.0016 | **2.9** | 0.9 |
| **Physical activity** (Ref: High) |  |  |  |  |  | **0.8** |  |  |  |  |  |  |  |
| Moderate | 0.0116 | 0.0049 | -0.2312 | -0.0011 | 1.2 | 0.6 |  | -0.0209 | -0.0151 | -0.1403 | 0.0021 | -4.0 |  |
| Low | 0.0447 | 0.0131 | -0.0257 | -0.0003 | 0.4 | 0.2 |  | 0.0173 | 0.0104 | 0.1073 | 0.0011 | -2.1 |  |
| **Body mass index** (Ref: Normal) |  |  |  |  |  | **28.7** |  |  |  |  |  |  |  |
| Undernutrition | -0.0965 | -0.0660 | 0.3377 | -0.0223 | 24.0 | 11.5 |  | -0.0722 | -0.0422 | 0.4613 | -0.0195 | 36.7 | 10.8 |
| Overweight | 0.1331 | 0.0637 | -0.3606 | -0.0230 | 24.7 | 11.9 |  | 0.0639 | 0.0582 | -0.1964 | -0.0114 | 21.5 | 6.4 |
| Obese | 0.0929 | 0.0197 | -0.5214 | -0.0103 | 11.0 | 5.3 |  | 0.2219 | 0.1670 | -0.3712 | -0.0620 | 116.7 | 34.4 |
| **Urban Residence** (Ref: Rural) | -0.0175 | -0.0328 | -0.4003 | 0.0131 | -14.1 |  |  | -0.0002 | -0.0005 | -0.3740 | 0.0002 | **-0.3** |  |
| **Regions** (Ref: Rift Valley) |  |  |  |  |  | **1.9** |  |  |  |  |  |  |  |
| Eastern | -0.0001 | 0.0000 | 0.0032 | 0.0000 | 0.0 | 0.0 |  | 0.0426 | 0.0331 | -0.0267 | -0.0009 | 1.7 | 0.5 |
| Nyanza | -0.0264 | -0.0114 | 0.0425 | -0.0005 | 0.5 | 0.3 |  | -0.0093 | -0.0053 | 0.0899 | -0.0005 | 0.9 | 0.3 |
| Coast | -0.0528 | -0.0252 | 0.0253 | -0.0006 | 0.7 | 0.3 |  | -0.0520 | -0.0246 | 0.0146 | -0.0004 | 0.7 | 0.2 |
| Central | 0.0185 | 0.0073 | -0.3171 | -0.0023 | 2.5 | 1.2 |  | 0.1064 | 0.0528 | -0.3381 | -0.0179 | 33.6 | 9.9 |
| Western | -0.0278 | -0.0105 | 0.0316 | -0.0003 | 0.4 | 0.2 |  | 0.0143 | 0.0050 | -0.0895 | -0.0005 | 0.8 | 0.3 |
| North Eastern | 0.1168 | 0.0165 | 0.6964 | 0.0115 | -12.4 |  |  | -0.0497 | -0.0142 | 0.6119 | -0.0087 | 16.4 | 4.8 |
| Nairobi | -0.0886 | -0.0047 | -0.4555 | 0.0021 | -2.3 |  |  | -0.0523 | -0.0031 | -0.6017 | 0.0019 | -3.5 |  |
| Concentration index (SE) |  | −**0.094 (**0.045**)** | | |  |  |  |  | −**0.053 (**0.036**)** | | |  |  |
| Residuals |  |  | 0.008 |  |  |  |  |  |  | −0.016 |  |  |  |
| 95% CI |  | −0.182 to −0.006 | | |  |  |  |  | −0.125 to −0.018 | | |  |  |

**Bold**: p <0.05. Coeff.: coefficient; C: concentration index; Cont. to C: contribution to concentration index; %: percentage contribution. SE: standard error; ** The absolute contribution of each determinant was divided by the total explained portion that make contributions to the same direction of the concentration index.
